# Intestinal Transcriptome Analysis Highlights Key Differentially Expressed Genes Involved in Nutrient Metabolism and Digestion in Yellowtail Kingfish (*Seriola lalandi*) Fed Terrestrial Animal and Plant Proteins

**DOI:** 10.3390/genes11060621

**Published:** 2020-06-05

**Authors:** Chinh Thi My Dam, Tomer Ventura, Mark Booth, Igor Pirozzi, Michael Salini, Richard Smullen, Abigail Elizur

**Affiliations:** 1 Genecology Research Centre and School of Science and Engineering, University of the Sunshine Coast, Maroochydore, DC 4558, Australia; Chinh.Dam@research.usc.edu.au (C.T.M.D.); tventura@usc.edu.au (T.V.); 2 Research Institute for Aquaculture No.1, Dinh Bang, Tu Son 220000, Bac Ninh, Vietnam; 3 New South Wales Department of Primary Industries, Port Stephens Fisheries Institute, Taylors Beach, New South Wales 2316, Australia; mark.booth@dpi.nsw.gov.au (M.B.); igor.pirozzi@dpi.nsw.gov.au (I.P.); 4 College of Science and Engineering & Centre for Sustainable Tropical Fisheries and Aquaculture, James Cook University, Townsville 4801, Australia; 5 Ridley Aquafeed Pty Ltd., 4/31 Robart Court, Narangba, Queensland 4504, Australia; michael.salini@ridley.com.au (M.S.); richard.smullen@ridley.com.au (R.S.)

**Keywords:** yellowtail kingfish, RNA-sequencing, transcriptomes, alternative protein sources, distal intestine, digestion

## Abstract

This study investigated the effects of dietary terrestrial animal and plant proteins on the intestinal transcriptomes of yellowtail kingfish (YTK), *Seriola lalandi*, an ecologically and economically important marine species in Australia. Five diets containing fish meal (FM), poultry by-product meal (PBM), blood meal (BLM), faba bean meal (FBM) and corn gluten meal (CGM) were formulated and fed over a period of 4 weeks. The Illumina RNA-sequencing (RNA-Seq) results identified a suite of differentially expressed genes involved in nutrient metabolism and protein digestion pathways, reinforced by quantitative polymerase chain reaction (qPCR) results. These findings provide molecular support to the notion that PBM and FBM are useful raw materials in commercial diets for YTK. Using the same evidence, we have demonstrated that BLM and CGM may be less useful and their incorporation into commercial aquafeeds for this species should be done cautiously. The differentially expressed genes showed a subtle difference and high correlation with apparent nutrient digestibility of raw materials. Further, our results indicate that transcriptome profiling provides a useful tool to evaluate alternative protein sources for use in aquaculture feeds.

## 1. Introduction

Yellowtail kingfish (YTK), *Seriola lalandi*, is an important marine carnivorous aquaculture species throughout the world due to its rapid growth and ravenous feeding behavior [1,2,3,4,5]. In Australia, YTK is commercially produced in sea cages in South Australia and Western Australia with small-scale trials conducted in New South Wales. The annual national production was estimated to be approximately 3,000 metric tons in 2017 and is expected to increase steadily in the next five years (David Head; Clean Seas Seafood, *personal communication*). With the expansion of the Australian YTK industry and the limited global supply of fish meal (FM), the future of YTK farming will therefore depend on alternative protein sources.

Over the last several years significant research has been conducted to identify suitable alternative protein sources that could fulfil the requirements of farmed fish [1,6,7,8,9,10,11]. Terrestrial animal by-products (e.g., poultry by-product meal (PBM), blood meal (BLM), meat and bone meal and feather meal etc.), contain significant levels of crude protein and provided they are processed correctly are usually well digested. As such rendered animal by-product meals have successfully been used to replaced FM in many fish diets [12,13,14,15]. Plant-based ingredients (e.g., soybean, dehulled field pea, wheat and corn gluten) have proved to be useful as partial or even total replacements of FM in both omnivorous and carnivorous aquaculture species [16,17,18,19,20,21]. However, the presence of anti-nutritional factors (ANFs) (e.g., saponins, lectins, protease inhibitors, oligosaccharides) in plant ingredients can cause a number of negative impacts on the digestive physiology, health and metabolism of fish resulting in reduced productivity [22,23,24]. Thus, for fish nutrition research, it is very important to evaluate the potential use of terrestrial animal and plant protein sources in the diet. 

The intestine is a multifunctional organ in fish. It is involved not only in feed digestion and absorption but also in pathogen recognition and regulation of the intestinal microflora [25,26]. In recent years, the number of transcriptomic studies in aquaculture has increased exponentially. Such studies have mainly used microarray platforms to investigate the effect of plant derived proteins on intestinal and liver transcriptomes. Tacchi et al. [27] found that genes involved in enteritis and protein and energy metabolism were up-regulated in the mid-intestine of Atlantic salmon, *Salmo salar,* fed a diet containing a combination of plant derived proteins (i.e., soy protein concentrate (SPC), corn gluten meal (CGM) and wheat gluten), compared to fish fed a diet high in FM. Recently research on *S. salar* reported that a mixture of SPC and bean protein concentrate (BPC) induced less extensive changes of intestine transcriptomes than diets made solely with either SPC or BPC [28]. Heat-treated of soybean meal removes the majority of ANFs, facilitating use of this product in rainbow trout, *Oncorrhynchus mykiss,* diets at a high inclusion level (50%) without causing enteritis or histological changes [29,30]. An experiment with European seabass, *Dicentrarchus labrax,* Reference [31] showed that plant protein sources both activate and suppress the expression of immune related genes. Murashita et al. [22] reported that Japanese yellowtail, *Seriola quinqueradiata,* fed a diet based on soybean meal and CGM displayed faster gastric emptying, lower pH of the gastrointestinal content and suppressed the expression of genes encoding pancreatic digestive enzymes (i.e., trypsin, chymotrypsin and amylase). To our knowledge, there are currently no reports on the use of RNA-sequencing technology to analyze the effect of both terrestrial animal and terrestrial plant protein sources on intestinal gene expression in YTK. 

The work presented here is a part of a larger body of work evaluating the impacts of alternative protein sources on apparent digestibility in YTK. Results regarding the nutrient digestibility of raw materials by YTK were reported elsewhere [32]. Our previous research found that YTK were efficient at digesting many commonly available raw materials including PBM, faba bean meal (FBM) and lupin kernel meal but were less efficient at digesting raw materials like BLM and CGM [32]. We hypothesized the low digestibility of BLM and CGM from our previous study was related to differentially expressed genes (DEGs). Therefore, the aim of the present study was to investigate the intestinal transcriptomes from YTK fed terrestrial animal and terrestrial plant proteins as dietary substitutes for FM. Specifically, our study aims to further understand the key genes involved in nutrient metabolism and digestion in YTK. RNA-Seq technology was utilized for nutrigenomic profiling and real-time quantitative polymerase chain reaction (qPCR) was used to analyze the changes of relevant genes encoding for key enzymes involved in nutrient metabolism and digestibility.

## 2. Materials and Methods 

### 2.1. Ethics

All experiments were performed according to Australian National Health and Medical Research Council (NH&MRC) animal ethics guidelines and regulations. Protocols were approved by the NSW Department of Primary Industries (NSW DPI) Fisheries Animal Care and Ethics Committee (Aquaculture Nutrition ACEC Authority 93/5-Port Stephens) and the Animal Ethics Committee of the University of the Sunshine Coast (AN/S/16/46).

### 2.2. Experimental Diets and Animals

The design of the digestibility experiments, protocols and composition of experimental diets discussed in the present study can be examined in detail in our previous study [32]. However, any information pertinent to the current study is provided herein (Supplementary file 1). Nonetheless, a brief overview of our previous work is provided here for clarity.

Two digestibility experiments were conducted with sub-adult YTK. Both experiments were conducted under the same conditions using the same fishmeal-based reference diet, the same pellet manufacturing procedures and the same experimental rearing system. There was no significant difference between the stocking weights of sub-adult YTK between two experiments, with YTK weighing 573.9 ± 17.6 g (mean ± SD) in the 1st experiment and 513 ± 17.1 g (mean ± SD) in the 2nd experiment [32]. The FM-based reference diet (68% prime FM) included yttrium oxide as the inert marker. Thirty percent of the reference diet was replaced with one of 14 feed ingredients (please see Reference [32] for complete list) in order to determine the apparent proximate and amino acid availability of each raw material. The one exception was BLM, which only replaced 15% of the reference diet to avoid the unpalatability at high inclusion. All diets were manufactured on a small-scale extruder and fed to sub-adult YTK in excess (2.5% of biomass) twice daily for 30 days. In this study, five diets including prime FM, PBM, BLM, FBM and CGM were selected for further analysis. Of the 5 diets examined, we adjudged the reference diet supplemented with additional FM to be the “benchmark diet” for YTK. We therefore consider the gene expression in fish fed the FM-based diet as “typical/the standard” of a healthy digestive system in sub-adult YTK. As such, all the responses of the intestine transcriptomes to PBM, BLM, FBM and CGM diets are expressed relative to those observed in fish fed the FM diet. The nutrient composition and apparent digestibility of these diets are presented in Supplementary file 1. Three experimental cages (*n* = 3), each housing six fish, were randomly assigned to each dietary treatment. During the digestibility trials, the average water temperature and dissolved oxygen concentrations were 20.3 ± 0.1 °C and 9.33 ± 0.3 mg L^−1^ respectively. The salinity ranged between 36 ppt to 37 ppt and the ambient photoperiod was 14 L:10D. Uneaten feed was collected and recorded on a daily basis to allow calculation of actual feed intake per experimental cage. Fecal samples were collected from sedated fish once per week using gentle stripping techniques. All fish were weighed at the beginning and end of the digestibility trials in order to assess specific growth rate (SGR) and feed conversion ratio (FCR) [33]; SGR (% body weight gain/day) = ((Ln (final weight (g))-Ln (initial weight (g))/30 days) × 100 FCR = total feed intake per experimental cage for 30 days (g as fed)/wet biomass gain per cage (g).

### 2.3. Tissue Sampling

Sub-adult YTK were fasted for 24 h before tissue samples were collected. Three fish were selected from eighteen fish in each dietary treatment (one fish per cage) and euthanized with AQUI-S (50–60 mg L^−1^AQUI-S, Melling, Lower Hutt, New Zealand, Ltd.). The fish were weighed and then quickly dissected in order to weigh the liver and viscera. The following morphometric parameters were calculated based on the wet weight of the fish and organs [33];

Hepatosomatic index (HSI) = (Liver weight/body weight) × 100

Viscerosomatic index (VSI) = (Viscera weight/body weight) × 100

Following this procedure, approximately 100 mg of the distal intestine was collected and placed in a tube with RNAlater (1.5 mL; Ambion) and stored at 4 °C for 24 h. These samples were then transferred to a −80 °C freezer until processed for sequencing analysis. Approximately 2 mm of the distal intestine was removed and fixed in a 10% neutral buffered formalin solution for 24 h, before transferring to 70% ethanol for storage prior to histological processing.

### 2.4. Histology

The distal intestine was processed in an automated tissue processor and infiltrated with paraffin according to standard methods. The intestinal tissue samples were sectioned at 5 µm and stained with haematoxylin and eosin (H&E). The slides were viewed and photographed using a Leica compound microscope (model DM5500 B, Leica Microsystems CMS GmbH, Ernst Leitz Strasse, Wetzlar, Germany). The morphology of the distal intestine was scored based on the lamina propria, mucosal folds and supranuclear vacuoles, with each parameter scored on a scale from 1 (no enteritis) to 5 (severe enteritis) [34].

### 2.5. RNA Extraction, Library Construction and High-Throughput Sequencing 

Total RNA was extracted from 30 mg of RNAlater stabilized intestinal tissue using a RNeasy Mini Kit (Qiagen, Melbourne, Victoria, Australia). Samples were disrupted and homogenized in 600 µL buffer RLT using a TissueRuptor (Qiagen, Melbourne, Victoria, Australia). RNA was extracted according to manufacturer’s instruction (Qiagen, Melbourne, Victoria, Australia). The quantity of total RNA was measured by Nanodrop spectrophotometry (ND-2000). The integrity of the total RNA was assessed using Agilent 2100 Bioanalyser (Agilent Technologies, Santa Clara, California, USA) with RNA Nano Chips and RNA 6000 Nano Assay Kit (Agilent Technologies, Santa Clara, California, USA). Samples were then stored at −80 °C for downstream analysis.

Quantitative sequencing libraries for the distal intestine were generated using Lexogen’s Quantseq 3” mRNA-Seq Kit (Lexogen, Vienna, Austria). Oligo dT primers were used to capture mRNA from Total RNA. After first strand synthesis, the RNA was removed and second strand synthesis was initiated with random primers containing an Illumina-compatible linker sequence and a DNA polymerase. All products were purified by a magnetic-based purification step and amplified by PCR for the generation of the pre-sequencing cDNA libraries. Each library was sequenced with read length of 100 bp on Illumina HiSeq 2500 at the Australia Genome Research Facility (AGRF, Melbourne, Victoria, Australia).

### 2.6. Bioinformatics

For differential expression (DE) analysis, CLC genomics workbench (Ver.11.01) was used. Raw reads were initially subject to quality filtering based on (1) discard reads with adaptor contamination, (2) discard reads when uncertain nucleotides constitute more than 10% of either reads (*n* > 10%) and discard reads when low quality nucleotides (base quality less than 20) constitute more than 50% of the reads. Quality-trimmed reads were then mapped to Yellowtail amberjack, *Seriola lalandi dorsalis* reference genome [35] as there is little speciation to warrant taxonomic distinction to *S. lalandi* [36] with the *map reads to reference* tool, using the following parameters: mismatches = 2; minimum fraction length = 0.9; minimum fraction similarity = 0.8 and maximum hits per read = 5 and then read counting was generated.

Differentially expressed genes (DEGs) between dietary treatments were identified by using DEseq2 false discovery correction rate (FDR) cut off = 0.05 and minimum fold change = 2. For the following analysis, DEGs were annotated by Blastx against non-redundant (nr) database with max blast hit 20, threshold of 10^−6^. The Blast results were loaded in Blast2Go software to obtain gene otology (GO) term for “biological process,” “cellular component” and “molecular function.” The Gene Set Enrichment Analysis (GSEA) method was used for pathway analysis.

### 2.7. Validation of RNA-Seq by qPCR

Quantitative PCR (qPCR) analysis was conducted to validate the reliability of the differential expression results from the RNA-Seq pipeline. The same RNA samples that were employed in the library preparation were also used for the qPCR analysis. Complementary DNA (cDNA) was synthesized by means of reverse transcriptase reaction using Tetro cDNA synthetic kit (Bioline, Australia) with 2 μg of total RNA. Gene specific primers and probes for genes of interest were designed from the species-specific mRNA sequences using Roche Assay Design platform [37]. 18S RNA was used as a reference gene for normalization and quantified by means of primers with the above-mentioned Master mix and probe. Primers and probes are detailed in Supplementary file 2.

qPCR was performed using a TOptical Thermocycler (Biometra, Germany) in ninety-six-well plates in duplicate 20 μL reaction volumes containing 10 μL of qPCR JumpStart Taq Master Mix (Sigma Aldrich, Australia), 2 μL and 0.2 μL of the primer (10 pmol) and probes corresponding to the analyzed gene respectively, 3.8 μL of molecular biology-grade water and 2 μL of cDNA. In addition, amplifications were carried out with a systematic negative control (non-template control) containing no cDNA. Standard amplification parameters contained an uracil-DNA glycosylase (UDG) pre-treatment at 50 °C for 2 min, an initial denaturation step at 95 °C for 10 min, followed by thirty-five cycles: 15 s at 95 °C, 30 s at the annealing temperatures and 30 s at 72 °C.

### 2.8. Statistical Analysis

Feed intake, growth and somatic indices are presented as means ± SD (*n* = 3). We have cautiously combined experimental data and analyzed it assuming no effect of the experiment, as data were obtained using the same batch of reference diet, the same cohort of YTK and the same experimental systems and protocols. Skewness/Kurtosis tests were used to test the normality of data. Significant differences in performance indices and qPCR were analyzed by one-way analysis of variance (ANOVA), followed by a post-hoc Tukey-range test. Differences were reported as statically significant when *p* < 0.05. Statistical analysis was performed using SPSS version 22.0 (SPSS, Michigan Avenue, Chicago, IL, USA). qPCR results were calculated using the Pfaffl method [36].

### 2.9. Data Deposition

RNA sequences were deposited in NCBI Sequence Read Archive (SRA) database with BioSample SAMN 14236812 (SRR 11196409 to SRR11196438) under BioProject PRJNA609208.

## 3. Results

### 3.1. Growth, Feed Intake and Somatic Indices

Sub-adult YTK fed the reference diet substituted with 30% FM recorded a numerically higher SGR compared to other treatments but there was no statistical difference in feed intake or SGR among dietary treatments (*p* > 0.05; Table 1). There were significant differences among the FCR of dietary treatments, with YTK fed FM and PBM having lower FCR (better) than YTK fed CGM or BLM, while the FCR of FBM was intermediate (*p* < 0.05; Table 1). Neither viscerosomatic (VSI) or hepatosomatic indices (HSI) were significantly affected by dietary treatment (*p* > 0.05; Table 1).

### 3.2. Histological Evaluation of the Distal Intestine

There were no significant differences found in the lamina propria, mucosal folds and supranuclear among sub-adult YTK fed diets containing the different raw materials after 4 weeks feeding (*p* > 0.05) (Table 1). A reduction in the length of mucosal folds and supranuclear vacuolization was noted in a single fish fed the diet containing CGM as compared to fish fed the diet containing FM but no inflammation signs were observed (Supplementary file 3).

### 3.3. Transcriptomic Analysis Overview

In total, 44,086,993 reads with a designated read length of 100 bp (single-end) were obtained from the distal intestine libraries. After sequence trimming, more than 90% of the reads remained. Mapping rate to the reference genome ranged from 58.05% to 98.42% (Supplementary file 4).

### 3.4. Differentially Expressed Genes (DEGs) in the Distal Intestine

**Figure 1:**
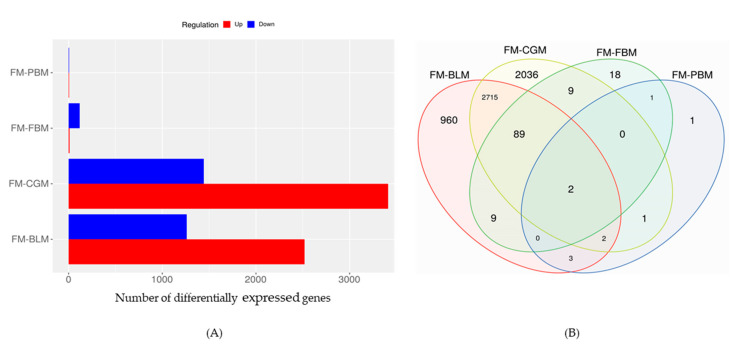
The number of differentially expressed genes (in the distal intestine of sub-adult Yellowtail kingfish fed a diet containing PBM, BLM, FBM or CGM relative to sub-adult YTK fed a diet composed predominantly of prime FM. (**A**) the number of differentially expressed genes between experimental diets and (**B**) Venn diagram showing the number of unique and shared gene cluster in 5 experimental diets. Abbreviations FM: fish meal, PBM: poultry by-product meal, BLM: blood meal, FBM: faba bean meal, CGM: corn gluten meal.

A total of 10 DEGs (i.e., 5 up/5 down) were observed in sub-adult YTK fed the diet containing PBM (Figure 1). A greater number of DEGs (3780) were observed in sub-adult YTK fed the diet containing BLM (i.e., 2519 up/1261 down). Sub-adult YTK fed the diet containing CGM recorded 4854 DEGs (i.e., 3413 up/1443 down), whereas only 128 DEGs (i.e., 10 up/118 down) were found in sub-adult YTK fed the diet containing FBM (Figure 1).

GO term analysis showed PBM and FBM did not induce significant enrichment categories while BLM and CGM affected the regulation of variety of genes involved in proteolysis, lipid metabolic processes, carbohydrate metabolic processes, oxidation-reduction and negative regulation of endopeptidase activity. The significantly changed GO terms are listed in Supplementary file 5.

**Figure 2:**
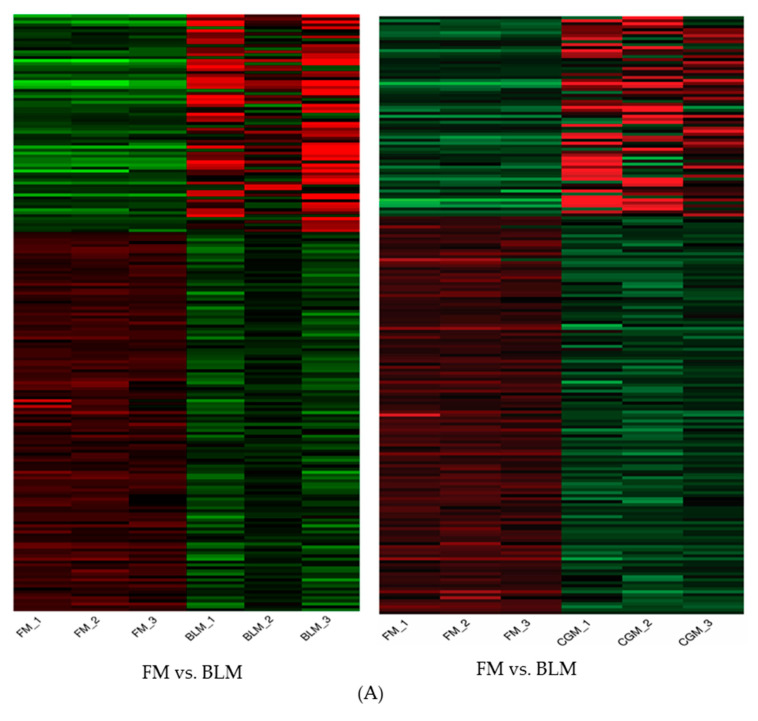

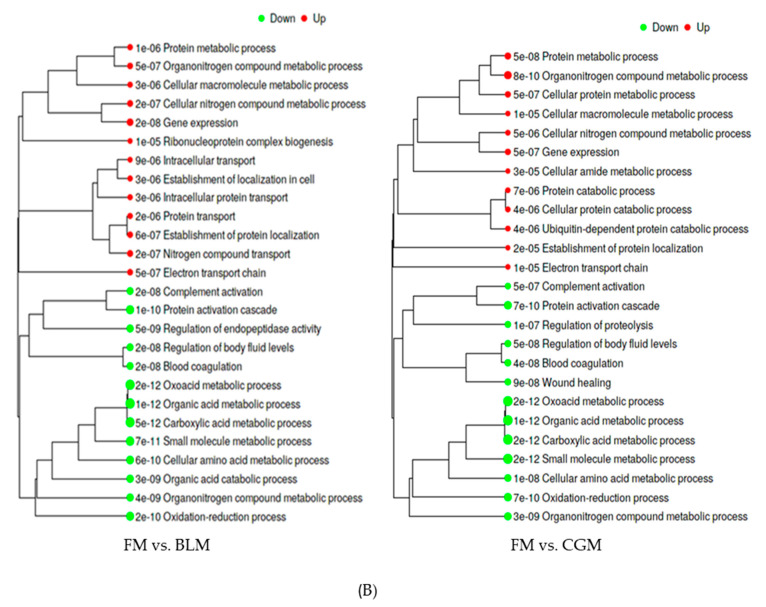
Enriched pathways in up and down-regulated genes of the 2 comparisons: FM vs. BLM and FM vs. CGM. (**A**) heatmaps (**B**) hierarchical clustering tree. Abbreviation: FM: fish meal, BLM: blood meal, CGM: corn gluten meal. Red: up-regulated pathways in FM group, Green: down-regulated pathway in FM group.

Pathways were assigned for DEGs in four diet comparisons, which were also analyzed using GSEA (FM diet vs. PBM diet, FM diet vs. BLM diet, FM diet vs. FBM diet and FM diet vs. CGM diet). In the case of the BLM and CGM diets, complement activation, regulation of proteolysis, regulation of endopeptidase activity and regulation of hydrolase activity were significantly enriched and up-regulated in the transcriptomes of sub-adult YTK. No KEGG pathway enrichment was observed in sub-adult YTK fed diets containing PBM or FBM. A heat map and hierarchical clustering tree depicting the DEGs related to the FM vs. BLM and FM vs. BLM comparisons are presented in Figure 2.

### 3.5. Expression of Key Nutrient Metabolism and Digestibility Related Genes

**Figure 3:**
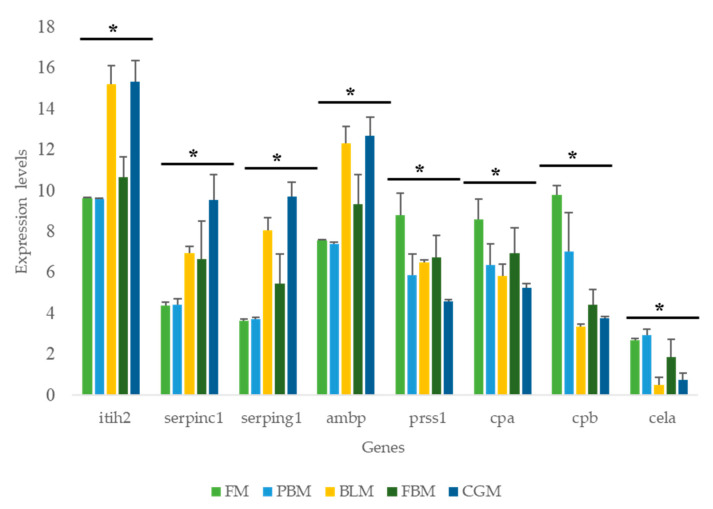
Expression levels of genes related to protein digestion in response to the different diets. Abbreviation: FM: fish meal, PBM: poultry by-product meal, BLM: blood meal, FBM: faba bean meal, CGM: corn gluten meal, *itih2*: trypsin inhibitor, *serpinc, serping 1*: serine protease inhibitor family: *ambp*: α-1 microglobulin/bikunin precursor, *prss1*: trypsin-1, *cpa*: cacboxypeptidase A, *cela*: chymotrypsin-like elastase, *cpb*: cacboxypeptidase B. Bars represent the mean of three biological replicates with their corresponding standard deviation. * indicate significant difference between two diets using t-test with *p* value < 0.05.

**Figure 4:**
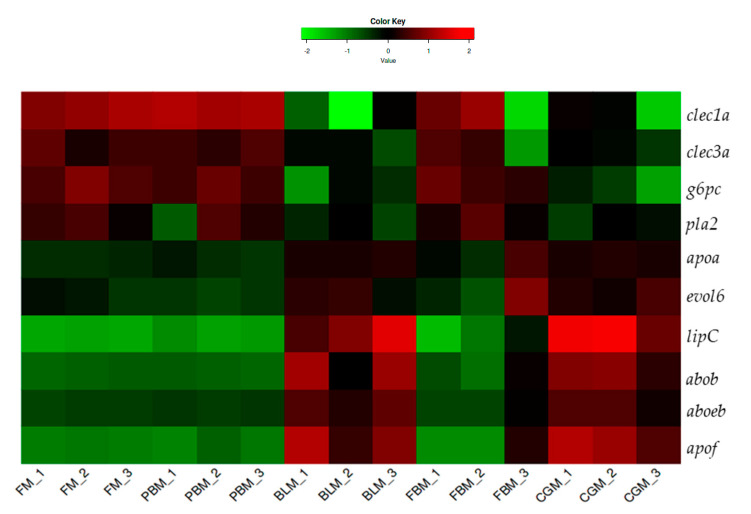
Expression levels of genes related to lipid and carbohydrates metabolism in fish fed different diets. Abbreviation: FM: fish meal, PBM: poultry by-product meal, BLM: blood meal, FBM: faba bean meal, CGM: corn gluten meal, *clec1a and clec3a*: C-type lectin domain family. *g6pc*: glucose-6-phosphatase, *pla2*: phospholipase A2, *apoa*: apolipoprotein A, *elovl 6*: elongation of very long chain fatty acid family 6, lipc: lipase C, *apob*: apolipoprotein B, *apoeb:* apolipoprotein Eb, *apof*: apolipoprotein f.

The RNA-Seq data showed that the expression of trypsin inhibitor (*itih2*), protease inhibitors (i.e., serine protease inhibitor family (*serpinc, serping1*) and α-1 microglobulin/bikunin precursor (*ambp*)) were up-regulated while genes encoding proteolytic digestive enzymes (i.e., trypsin (*prss1*), cacboxypeptidase A (*cpa*), cacboxypeptidase B (*cpb*) and chymotrypsin-like elastase (*cela*)) were down-regulated in sub-adult YTK fed either BLM or CGM compared to fish fed FM (Figure 3). The expression of genes related to protein metabolism (i.e., solute carrier family member 1 (*scl1*), solute carrier family member 3 (*scl3*), solute carrier family member 5 (*slc 5*), solute carrier family member 7 (*slc7*), solute carrier family member 9 (*slc 9*), cathepsin L (*ctsL*) and cathepsin K (*ctsK*)) were generally higher in sub-adult YTK fed diets containing BLM and CGM (Supplementary file 6). The DEGs involved in lipid and carbohydrate metabolism are presented in Figure 4. Diets containing BLM and CGM modulated a higher number of up-regulated genes encoding proteins involved in lipid metabolism (i.e., lipase C (*lipC*), phospholipase A2 (*pla2*), elongation of very long chain fatty acid family 6 (*elovl 6*), apolipoprotein A (*apoa*), apolipoprotein B *(apob*) apolipoprotein Eb, (*apoeb*) and apolipoprotein f (*apof*)) and generally down-regulated genes involved in carbohydrate metabolism (i.e., glucose-6-phosphatase (*g6pc*) and C-type lectin domain family (*clec1, clec3*)).

### 3.6. qPCR Validation

**Figure 5:**
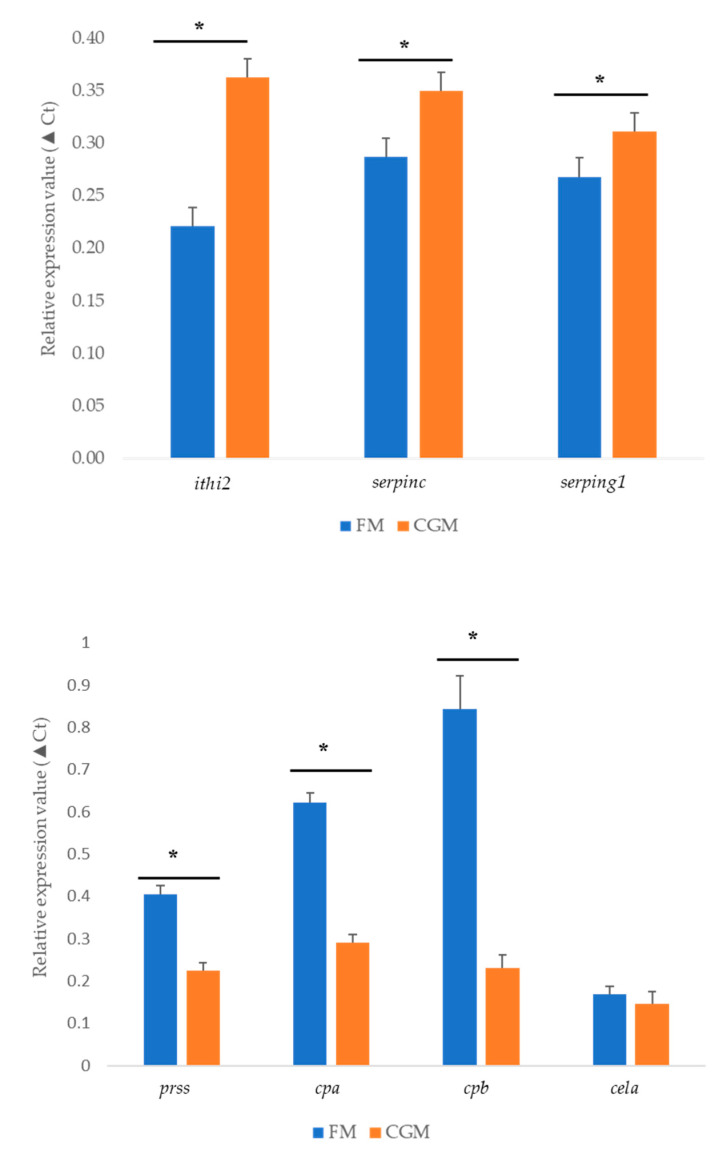
Quantitative polymerase chain reaction (qPCR) validation for 7 genes related to protein digestion. Abbreviation: FM: fish meal, CGM: corn gluten meal, *itih2*: trypsin inhibitor, *serpinc, serping 1*: serine protease inhibitor family: *prss1*: trypsin-1, *cpa*: cacboxypeptidase A, *cela*: chymotrypsin-like elastase, *cpb*: cacboxypeptidase B. * indicates a significant difference between two diets with *p* value < 0.05.

The RNA-Seq results point to differential expression of the proteolytic digestive enzymes and inhibitors in the YTK intestine. Based on the apparent nutrient digestibility values of raw materials that we obtained from our previous study [32], we selected 4 genes encoding digestive enzymes (*prss1, cpa, cpb cela*) and 3 genes related to inhibitors (*ithi2, serpinc, serping1*) for qPCR validation in the distal intestine tissue of sub-adult YTK fed the FM diet (i.e., control) and the least digestible diet (i.e., diet containing 30% CGM). The expression pattern of those genes was consistent with the results from the RNA-Seq (Figure 5). ▲Ct value qPCR and log2 transformed expression values from RNA-Seq showed significant correlation, however, the correlation was weak (R^2^ = 0.34, *p* < 0.05, Supplementary file 7).

## 4. Discussion

To date, little is known about the effect of replacing FM with alternative protein sources on the fish transcriptome and importantly, how that relates to nutrient digestibility or availability. In our examination, we used intestinal tissue collected from sub-adult YTK that were fed experimental diets containing relatively high levels of either plant or animal meal. These diets were originally formulated to determine the apparent digestibility of individual raw materials that might be used in commercial aquafeeds for this species [32]. Experimental diets like these are unlikely to be used commercially due to the extreme content of individual raw materials (i.e., up to 30%). However, the very nature of these diets offers a unique opportunity to examine the effect of novel raw materials on the digestive transcriptome of fish and help explain why some raw materials are poorly digested and therefore unsuitable for use in aquafeeds at high inclusion levels. To our knowledge, the present study is among the first to demonstrate that alternative raw materials affect gene expression in sub-adult YTK and that the regulation of certain genes encoding for digestive enzymes correlate with the poor digestibility of certain raw materials. Our study assumes that sub-adult YTK fed a diet composed primarily of high-quality FM reflect levels of gene expression typical of a healthy digestive system in this species (i.e., at least of a farmed animal) [28] and the expression of genes in response to other dietary treatments will vary relative to that found in fish conditioned to the FM diet. Our major findings indicate that alternative protein sources such as BLM (15% of diet) and CGM (30% of diet) had a significant impact on distal intestine transcripts related to nutrient metabolism and digestion by down-regulating genes encoding for digestive enzymes and up-regulating genes encoding for proteinase inhibitors, which may partly explain the poor apparent nutrient digestibility of these raw materials [32].

Based on the anatomical scores used in our study, we found no differences in the histological sections of the distal intestine of fish after 4 weeks feeding on different dietary treatments. Visual signs of inflammation were also absent. Similarly, little or no change has been observed in histological samples taken from the foregut or hindgut of YTK fed soybean meal and soy protein concentrate, albeit the results tend to vary depending on which section of the intestine is examined, especially with respect to goblet cell proliferation and supranuclear vacuolization [38]. Nonetheless minor changes to the gut were expected, particularly in fish fed the FBM and CGM diets, as the feeding of plant proteins such as soybean meal [39] and soy protein concentrate has been implicated in low digestibility [40] and poor gut health in YTK [41]. The reasons why no anatomical changes were observed remains unclear but it may relate to the short duration of feeding (30 days). To some extent, the fish were already exposed to commercial feeds and raw materials due to their previous holding conditions, so the gastro-intestinal tract may already be pre-conditioned to these types of feeds; hence the lack of anatomical changes. It might also be attributed to the type of processing applied to each of the raw materials (which is unknown) prior to their inclusion in our test diets or the cooking of diets during the extrusion manufacturing of our pellets. These processes are well known to reduce several anti-nutritional factors (ANFs) in plant proteins, especially legumes that can affect or inhibit digestibility and also lead to enteritis like conditions [42,43,44]. Therefore, further ANFs analysis of the raw materials and longer-term experiments are needed to confirm the lack of histological observations. Some changes were observed in a single fish fed the diet containing CGM, including shortening of mucosal folds and a decreased number of absorptive vacuoles in the enterocytes, however no significant differences were found among YTK fed the experimental diets. Other authors have speculated that the low digestibility of CGM in *Seriola* species such as *S. quinqueradiata* might be due to the low pH of CGM (pH ≈ 3.2), which may interfere with amino acid availability [45]. Interestingly, no significantly differentially expressed transcripts involved in inflammation were found in the RNA-Seq results, which support the lack of histological abnormality in this study. This result contrasts with studies on California yellowtail, *S. dorsalis* [23], Atlantic salmon, *S. salar* [28,46] and turbot, *Scophthalmus maximus* [47]. The differences with our finding might be related to the longer trial duration in those studies (56 days to 96 days) or the varying response in different species.

Our major focus in this study, was to investigate the transcriptional response to different and extreme diets in the distal intestine, a multifunctional tissue for nutrient uptake, pathogen recognition and regulating the intestine microflora. The transcription of genes involved in the metabolism of proteins, lipids and carbohydrates in the intestine of sub-adult YTK were modulated significantly among the experimental diets, showing rapid response and adaptation of the distal intestine molecular machinery to the different nutritional conditions. Furthermore, the nutritional composition of the five experimental diets differed and were formulated using different protein sources, which together might be metabolically challenging to YTK. The differentially expressed transcripts related to nutrient metabolism are consistent with those reported for Atlantic salmon, *S. salar* [27,28], European seabass, *D. labrax* [31,48] and rainbow trout *O. mykiss* [49,50], where dietary components also changed the expression of genes related to primary metabolic functions (e.g., lipid, amino acid and energy) in those species. In our trial, the distal intestine displayed a strong transcriptomic response, confirming the sensitivity of intestinal cells to dietary changes.

The diets containing BLM and CGM showed the highest DEGs in the distal intestine, with PBM and FBM showing a lower response, as compared to the diet composed of prime FM. The up-regulation of genes encoding proteolytic digestive enzymes (*prss1, cela, cpa, cpb*) was high in the YTK intestine, indicating that these genes play a crucial role in digestive functions. *Prss1* is considered to be the most important proteolytic enzyme, as it plays a key role in hydrolyzing proteins and activating other digestive zymogenes [22,51,52]. *Cela, cpa* and *cpb* are involved in protein digestion [51]. In this study, both RNA-Seq data and qPCR results (except *cela*) show that fish fed the diet containing CGM, had a significant down-regulation of genes encoding proteolytic digestive enzymes, suggesting that dietary proteins were not being efficiently digested, possibly due to the low activity of these enzymes in the gut and or distal intestine. This result is entirely consistent with the low and erratic digestibility data presented for CGM in our previous study [32]. These results agree well with the results from a published study [22] on the Japanese yellowtail, *S. quinqueradiata*, where the expression levels of all the digestive enzyme genes were lower in a diet replacing FM with SBM and CGM. Interestingly, our study found that genes related to endopeptidase inhibitor activity (i.e., *itih2, serpinc, serping1*) were overexpressed in the intestine of sub-adult YTK fed diets containing BLM and CGM, which suggests that the digestive capacity of the fish was inhibited, consistent with the high protein content in the fecal matter of fish fed these two diets [32]. It is therefore possible that enzyme inhibitors present in BLM and CGM diets may be a contributing factor responsible for the reduced digestibility of these particular diets. Intestinal tissue has an extremely high rate of cellular turnover and thus generally high levels of protein metabolism. Cathepsins are lysosomal cysteine proteases, which play crucial metabolic roles in maintenance of cellular homeostasis in organisms [53,54]. Our RNA-Seq results revealed an up-regulation of certain number of cathepsins related to protein synthesis and degradation (*i.e., ctsL, ctsK, slc family member*) by the dietary alternatives (except PBM), suggesting an increase in the intestinal protein turnover in fish fed those diets. These findings agree with a previous study on Atlantic salmon, *S. salar* [27], the authors found the up-regulation of *ctsZ* and other cathepsins in the fish fed a plant-based diets. Recently, in a study where rainbow trout, *O. mykiss,* were fed a diet containing plant protein sources, the results revealed differential regulation of *ctsZ* and *ctsH*, genes involved in protein catabolism [50]. Both protein synthesis and protein degradation are highly energy demanding processes [55], the high protein turnover in fish fed alternative protein sources (except PBM) is related to the overexpression of oxidative energy metabolism.

Previous studies reported that alternative protein sources could influence lipid metabolism and transport [28,56,57]. In our study, we also investigated DEGs related to fatty acid biosynthesis, phospholipid transport and digestion, including *lipC, pla2, elovl 6, apoa, apoeb* and *apof.* Lipase is a key enzyme that catalyzes the hydrolysis of lipids [58]. Apolipoprotein (*apoa, apob, apoeb, apof*) are lipid-associated proteins, that regulate lipid homeostasis by mediating triacylglycerol and phospholipid transport from the liver to other tissues [59,60]. Our RNA-Seq result showed an overexpression of these lipid metabolism-related genes in the transcriptomes from the dietary alternatives in response to the decrease of dietary lipid content (with the exception of PBM) (Supplementary file 1), which suggests an increased requirement for lipid in YTK fed these diets. These data are consistent with previous studies on Atlantic salmon, *S. salar* [27,28]. In terms of carbohydrate metabolism, *g6pc* and *celc* were under-expressed in alternative plant protein sources, suggesting that the mRNA and the levels of glycerol and glucokinase in the intestine of YTK respond to the proportion of carbohydrate in the diets [61], suggesting YTK has not adapted to efficiently use dietary carbohydrates as a major energy substrate [1]. Our observation on carbohydrate adaptation also agrees with studies on Atlantic salmon, *S. salar* [56] and gilthead seabream, *S. aurata* [62,63].

Based predominantly on transcriptomic evidence obtained from intestinal samples collected from sub-adult YTK used in a digestibility experiment, we demonstrate that PBM and FBM have potential to be useful raw materials in commercial diets for YTK. Using the same evidence, we have demonstrated that BLM and CGM may be less useful and their incorporation into commercial aquafeeds for this species should be done cautiously. The distal intestine transcriptome profiles revealed that a large number of DEGs were affected by the alternative protein sources we examined. A series of DEGs involved in protein, lipid and carbohydrate metabolism were identified. Furthermore, qPCR results highlighted the agreement between the RNA-Seq data for the down-regulated genes encoding digestive enzymes (*prss1, cpa, cpb, cela*) and the up-regulated genes encoding protease inhibitors (*itih2, serpinc, serping1*). Our results correlate well with observations on apparent digestibility and indicate that transcriptome profiling provides a useful tool to evaluate alternative protein sources in YTK and may be a useful approach in other commercial species.

## Figures and Tables

**Table 1:** Growth performance and histology parameters.

	FM	PBM	BLM	FBM	CGM
Initial weight (g)	586.33 ± 20.55	568 ± 12.52	517 ± 25.16	580.33 ± 8.62	561.67 ± 30.24
Harvest weight (g)	939.33 ± 83.16	789 ± 11.27	722.33 ± 49.94	856.33 ± 61.43	780.67 ± 85.70
SGR (%/day)	1.56 ± 0.29	1.10 ± 0.09	1.11 ± 0.33	1.29 ± 0.24	1.09 ± 0.22
Feed intake (g/day)	79.93 ± 3.73	80.07 ± 3.06	81.03 ± 2.85	79.23 ± 4.77	82.87 ± 6.63
FCR	1.65 ± 0.07 ^a^	1.83 ± 0.19 ^a^	2.53 ± 0.30 ^b^	2.25 ± 0.18 ^ab^	2.49 ± 0.40 ^b^
VSI (%)	3.94 ± 0. 15	4.03 ± 0.23	3.94 ± 0.06	3.94 ± 0.12	4.05 ± 0.13
HSI (%)	0.79 ± 0.11	0.74 ± 0.06	0.84 ± 0.07	0.86 ± 0.15	0.83 ± 0.13
**Histological score**					
Lamina propria	1.33 ± 0.57	1 ± 0.57	1.33 ± 0.57	1.33 ± 0. 57	1.67 ± 1.15
Mucosal folds	1.67 ± 0.57	1.67 ± 0.57	2.00 ± 0.00	1.67 ± 0.57	2.00 ± 0.00
Supranuclear vacuoles	1.33 ± 0.57	1.67 ± 1.15	2.00 ± 0.00	2.00 ± 0.00	2.33 ± 0.57

Abbreviations: FM, fish meal; PBM: poultry by-product meal; BLM: blood meal; FBM: faba bean meal CGM: corn gluten meal. Specific growth rate (SGR), apparent feed intake, feed conversion ratio (FCR), viscesomatic indices (VSI), hepatosomatic, (HSI) and histological scores of sub-adult Yellowtail kingfish fed a fish-meal based reference diet or a reference diet mixed with a single raw material. Values are mean ± SD of triplicate groups (number of cages in RAS; *n* = 3). Different superscript letters in the same row denote the differences (*p* < 0.05) determined by one-way ANOVA.

## Supplementary Materials

The following are available online at https://www.mdpi.com/2073-4425/11/6/621/s1, Supplementary file 1: Formulation nutrient composition and digestibility values of experimental diets, Supplementary file 2: The details of primers and probes used in this study, Supplementary file 3: Distal intestine histology of fish (hematoxylin and eosin) of fish fed experimental diets, Supplementary file 4: Number of reads and mapping rates among the samples, Supplementary file 5: Significant GO terms among treatments, Supplementary file 6: The expression of genes related to protein metabolism, Supplementary file 7: Correlation analysis of RNA-Seq and qPCR data of differentially expressed genes involved in digestion.
