# Supplementary material for: Intestinal Transcriptome Analysis Highlights Key Differentially Expressed Genes Involved in Nutrient Metabolism and Digestion in Yellowtail Kingfish (Seriola lalandi) Fed Terrestrial Animal and Plant Proteins
Source: Genes (Basel). 2020 Jun 5;11(6):621. doi: 10.3390/genes11060621 (PMC7349653; doi:10.3390/genes11060621)
Supplement: Supplementary file 1 [file genes-11-00621-s001.zip › supplementary/Supplementary file 1.docx]

Supplementary 1: The formulation, nutritional composition (g kg^-1^ or MJ kg^-1^ dry matter basis) and nutrient digestibility values (mean ± SD) (%) of experimental diets

|  | **FM** | **PBM** | **BLM** | **FBM** | **CGM** |
| --- | --- | --- | --- | --- | --- |
| Reference mash ^*^ | 700 | 700 | 850 | 700 | 700 |
| Fishmeal prime quality (FM) | 300 |  |  |  |  |
| Poultry by product meal (PBM) |  | 300 |  |  |  |
| Blood meal (BLM) |  |  | 150 |  |  |
| Faba bean meal (FBM) |  |  |  | 300 |  |
| Corn gluten meal (CGM) |  |  |  |  | 300 |
| **Nutrient composition (g kg^-1^)** |  |  |  |  |  |
| Crude protein | 605.9 | 598.6 | 635.1 | 507.7 | 603.7 |
| Ash | 144 | 135.2 | 109.9 | 98.9 | 94.5 |
| Lipid | 76.5 | 99.7 | 69.1 | 48.9 | 70.8 |
| NFE ^**^ | 173.6 | 166.6 | 185.9 | 344.5 | 230.9 |
| Gross energy (MJ kg^-1^) | 20.6 | 21.1 | 21.4 | 20.3 | 21.7 |
| **Nutrient digestibility value (mean ± SD) (%)** |  |  |  |  |  |
| Dry matter | 46.4 ± 1.7 | 46.3 ± 0.8 | 41.8 ± 1.4 | 40.2 ± 0.6 | 31.7 ± 2.9 |
| Protein | 66.4 ± 2.7 | 65.6 ± 0.8 | 56.8 ± 1.1 | 72.9 ± 2.0 | 50.3 ± 3.6 |
| Lipid | 87.4 ± 2.0 | 82.1 ± 0.9 | 80.6 ± 0.7 | 81.4 ± 1.6 | 63.3 ± 2.4 |
| Gross energy | 62.2 ± 1.9 | 61.4 ± 2.6 | 50.8 ± 1.4 | 61.0 ± 1.0 | 39.7 ± 2.5 |
| **Amino acid composition**  **(g kg^-1^)** |  |  |  |  |  |
| Alanine | 36.8 | 35.2 | 38.7 | 27.8 | 40.7 |
| Arginine | 30.5 | 30.1 | 28.6 | 27.1 | 24.9 |
| Aspartic acid (+ asparagine) | 58 | 53.9 | 59.8 | 48.6 | 50.9 |
| Cysteine | 4.5 | 4.7 | 4.9 | 4 | 5.8 |
| Glutamic acid (+ glutamine) | 86.4 | 83.7 | 84.1 | 75.2 | 103.7 |
| Glycine | 38.9 | 43.1 | 36.5 | 29.9 | 31.2 |
| Histidine | 10.6 | 8.9 | 12.8 | 7.8 | 8.9 |
| Isoleucine | 21.5 | 19.3 | 17 | 16.6 | 20.1 |
| Leucine | 41.9 | 39.1 | 48.3 | 34.3 | 57 |
| Lysine | 41.9 | 36.4 | 43.4 | 32 | 29.1 |
| Methionine | 16.4 | 13.5 | 14.1 | 11.3 | 14.7 |
| Phenylalanine | 24.7 | 23 | 29.1 | 20.1 | 28.3 |
| Proline | 27.4 | 30.4 | 27.8 | 22.9 | 37.1 |
| Serine | 30 | 29.4 | 31.8 | 25.2 | 31.3 |
| Taurine | 14.7 | 13.5 | 15.2 | 12.7 | 12.5 |
| Threonine | 26.5 | 24.8 | 27.8 | 20.6 | 24 |
| Tyrosine | 21.1 | 19.6 | 20.9 | 17 | 24.9 |
| Valine | 28.5 | 26.6 | 32.8 | 22.7 | 27 |
| **∑ reported amino acids + taurine** | **560.2** | **535.7** | **573.6** | **455.8** | **572.1** |
|  |  |  |  |  |  |

* Reference mash: FM (68%), wheat flour (27%) and fish oil (3.5%) as well as a vitamin / mineral premix (0.66%).

** NFE**:** Nitrogen free extract (NFE) calculated by difference. NFE = 1000- (protein + fat + ash)
