# Supplementary material for: Intestinal Transcriptome Analysis Highlights Key Differentially Expressed Genes Involved in Nutrient Metabolism and Digestion in Yellowtail Kingfish (Seriola lalandi) Fed Terrestrial Animal and Plant Proteins
Source: Genes (Basel). 2020 Jun 5;11(6):621. doi: 10.3390/genes11060621 (PMC7349653; doi:10.3390/genes11060621)
Supplement: Supplementary file 1 [file genes-11-00621-s001.zip › supplementary/Supplementary file 2.docx]

| Gene name (symbol) | Sequences (5^’^-3^’^) | T­_m_ | Universal ProbeLibrary Number | Product size (bp) |
| --- | --- | --- | --- | --- |
| Housekeeping gene (18S) | F: aggactccggttctattttgtg | 60 | 57 | 60 |
|  | R: cggccgtccctcttaatc | 60 |  |  |
| Trypsin (prss) | F: cctggtcaacgagaactgg | 59 | 84 | 60 |
|  | R: ggagtgacatacgacttgtagca | 59 |  |  |
| Cacboxypeptidase a (cpa) | F: gctcccagtactcccaacaa | 60 | 40 | 63 |
|  | R: acactgctcattggacagca | 60 |  |  |
| Carboxypeptidase b (cpb) | F: gcgtgacactggtcgttatg | 60 | 27 | 60 |
|  | R: tcacatgtgggcttgatctg | 60 |  |  |
| Chymotrypsin- elastase (cela) | F: cggcagataactggctaagg | 59 | 111 | 70 |
|  | R: ccatgcagtgagaagtaggatg | 59 |  |  |
| Trypsin-inhibitor (ihit2) | F: catgtgaaataaggtatcactgttca | 59 | 2 | 95 |
|  | R: ctcttcttggcggctgtc |  |  |  |
| Protease inhibitor (serpinC) | F: ggggaagaggaaggaaaggt | 60 | 14 | 76 |
|  | R: ccaccagcatgttacctgttt | 59 |  |  |
| Protease inhibitor (serping1) | F: acatctttttctcacctctgagc | 59 | 22 | 64 |
|  | R: ccgtcagaagccattacgag | 60 |  |  |

Supplementary 2: Sequence of primers and probes used in present study
