# Supplementary material for: Intestinal Transcriptome Analysis Highlights Key Differentially Expressed Genes Involved in Nutrient Metabolism and Digestion in Yellowtail Kingfish (Seriola lalandi) Fed Terrestrial Animal and Plant Proteins
Source: Genes (Basel). 2020 Jun 5;11(6):621. doi: 10.3390/genes11060621 (PMC7349653; doi:10.3390/genes11060621)
Supplement: Supplementary file 1 [file genes-11-00621-s001.zip › supplementary/supplementary file 7.docx]

Supplementary file 7: Correlation analysis of RNA-Seq and qPCR data of differentially expressed genes involved in digestion
